# Supplementary material for: MetaRibo-Seq measures translation in microbiomes
Source: Nat Commun. 2020 Jun 29;11:3268. doi: 10.1038/s41467-020-17081-z (PMC7324362; doi:10.1038/s41467-020-17081-z)
Supplement: Supplementary file 10 — Supplementary Data 7 [file 41467_2020_17081_MOESM10_ESM.zip › File2/Confidence_High_Taxonomy/233103_out.krona.html]

Javascript must be enabled to view this page.

members
magnitude
magnitudeUnassigned
count
unassigned
taxon
rank

233103\_out

2

superkingdom
2
2

2
1239
phylum

class
186801
2

order
2
186802

family
541000
2

1
1898205

SRS1041031\_contig\_number\_contig-100\_25840.58741
species

1
1263
genus


SRS078176\_contig\_number\_9892
species
1
1262959
